# Diabetic macular oedema (DMO): an introduction

**Published:** 2025-01-31

**Authors:** Rajiv Raman, Raja Narayanan

**Affiliations:** 1 Senior Consultant: Shri Bhagwan Mahavir Vitreoretinal Services, Sankara Nethralaya, Chennai. India; 2 Professor of Practice: Department of Medical Science and Technology, IIT-Madras, Chennai, India; 3 Visiting Professor : Vision and Eye Research Unit, Anglia Ruskin University, Cambridge, UK; 4 Director: Anant Bajaj Retina Institute, L.V. Prasad Eye Institute, Hyderabad, India.

**DMO is a complication of diabetes that requires a collaborative approach for both early detection and long-term management.**

The number of people affected by diabetes is continuing to rise worldwide. Diabetes causes high blood sugar levels, which damages blood vessels and nerves in various tissues, resulting in complications such as cardiovascular disease, kidney disease, foot problems (sometimes resulting in amputation), and damage to the retina, known as diabetic retinopathy.

Diabetic macular oedema (DMO) is a complication of diabetic retinopathy and occurs when vascular endothelial growth factor (VEGF) and other inflammatory factors alter the blood-retinal barrier. This causes leakage of fluid around the macula – the area responsible for central vision – causing retinal thickening and exudation. The resulting distortion and reduction in central vision can profoundly impact people's quality of life, restricting key daily activities.

DMO is the most common cause of sight loss in people with diabetes. Key risk factors include prolonged diabetes duration, poor blood sugar control, high blood pressure, and hyperlipidemia.^1^

## Community/primary level

Suspect that a patient has DMO if:

- they have diabetes
- they report loss of vision
- they report distortion of vision
- they have other complications of diabetes, such as foot damage

Primary care providers should refer patients with suspected DMO for an eye examination by an ophthalmologist.

## Clinical examination

Diabetic macular oedema can be detected in different ways, depending on the personnel and equipment available.

1. **Dilated fundus examination.** Using a direct ophthalmoscope, perform a careful fundus examination through dilated pupils to inspect the retina for signs of diabetic retinopathy and macular oedema. Dilating the pupil is essential, as it allows examination of the macula, enabling the detection of retinal thickening, hard exudates, and other signs indicative of DMO.
2. **Slit lamp biomicroscopy with a handheld lens.** This method allows detailed, and three-dimensional, visualisation of the macula using a slit lamp and a 90D or 78D lens. This technique is ideal for identifying changes in the macula and subtle signs of diabetic macular oedema.
3. **Fundus photography.** Photographing the retina to detect and document changes over time.
4. **Telemedicine and remote screening tools.** In resource-limited settings, teleophthalmology can be used to examine patients remotely. Images captured by non-mydriatic cameras in primary care centres can be sent to a specialist for evaluation.
5. **OCT.** OCT is valuable for detecting and quantifying retinal thickening and fluid accumulation characteristic of DMO.

## Clinical features

**Figure 1:**
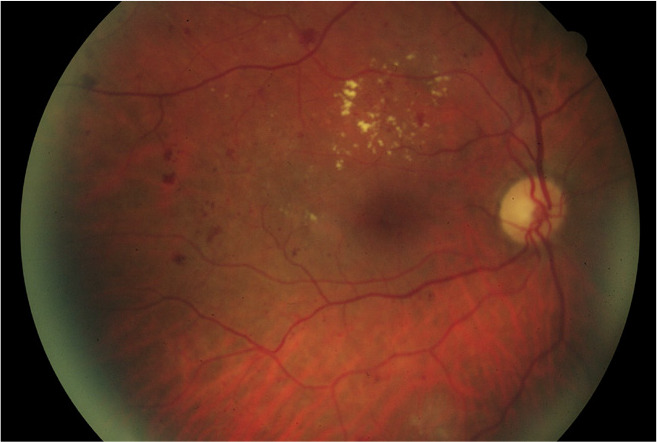
Mild DMO

**Figure 2:**
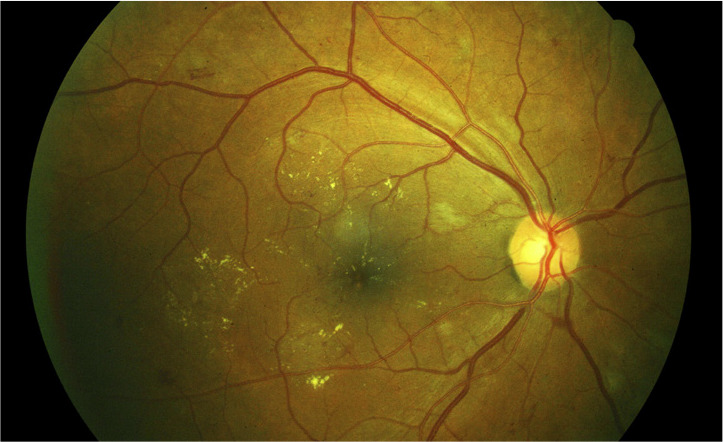
Moderate DMO

**Figure 3:**
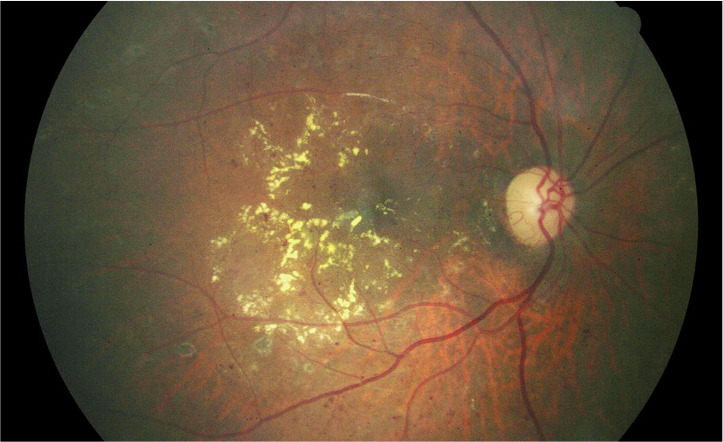
Severe DMO

Diabetic macular oedema is characterised by retinal thickening and the presence of hard exudates. The severity is categorised as mild (Figure 1), moderate (Figure 2), or severe (Figure 3) based on the location and extent of these lesions. Mild DMO features retinal changes more than two thirds of a disc diameter (1,000 μm) from the central macula. Moderate DMO involves lesions close to but not involving the fovea, whereas severe DMO includes lesions that affect the fovea, posing a significant threat to central vision.^2^

© DAVID YORSTON CC BY-NC-SA 4.0

© DAVID YORSTON CC BY-NC-SA 4.0

© DAVID YORSTON CC BY-NC-SA 4.0

Patients with moderate or severe diabetic macular oedema should be referred to an eye specialist, who will classify DMO into centre-involving and non-centre-involving DMO using clinical criteria. Centre-involving DMO generally requires active treatment, such as intravitreal injections using anti-VEGF drugs (see page 10 in this issue). In contrast, non-centre-involving DMO may be managed by observation and reducing the risk of progression through improved control of blood sugar and blood pressure. For some patients, laser treatment may be useful, if it is available.

Regular follow-up is critical to monitor DMO and to adjust treatment as necessary.

## Patient education and self-monitoring

Educating patients with diabetes about the early symptoms of DMO is crucial. Common symptoms to monitor include blurred vision or distorted vision, where straight lines may appear wavy or bent. Additionally, colours may appear faded or washed out, and dark spots or blank areas might obscure the central field of vision.

Patients should be encouraged to monitor their own vision using tools like the Amsler grid (if available) or by looking at a straight doorway or window frame to help detect visual distortions early. They should report any changes in vision to their healthcare provider promptly. If a screening programme exists, all people with diabetes should be encouraged to take advantage of this. Retinal photography is effective at detecting DMO before any sight is lost, and early detection and treatment leads to better outcomes.

## Screening, referral and effective management

Diabetic retinopathy, especially in adults, is a slowly progressive condition. Typically, it takes decades after detecting the early signs of diabetic retinopathy before someone develops sight-threatening diabetic retinopathy, including diabetic macular oedema. Screening people with diabetes for early signs of retinopathy means that there is time to act before vision impairment develops.

A strong referral system is crucial. Eye health workers should know when, where, and how to refer patients who have potentially sight-threatening retinopathy. Ophthalmologists who are able to treat diabetic retinopathy should ensure that all local clinics are aware of the service offered, and understand the referral pathway.

Effective management of DMO depends on collaboration between primary care providers and eye care specialists. Clear communication and coordination will ensure patients receive timely and appropriate care.

## Risk factor management and prevention

Effective management of DMO involves addressing systemic conditions that exacerbate the condition. Apart from good control of blood sugar, other interventions may help to control or prevent DMO. Anaemia accelerates diabetic complications and DMO progression and should be managed. Dyslipidemia (high blood cholesterol) significantly contributes to DMO, and lipid-lowering drugs like fenofibrate have been shown to reduce DMO severity irrespective of actual lipid levels.^3^ Managing renal disease is crucial, as nephropathy correlates strongly with DMO outcomes, influencing treatment responses.^4^

Cardiovascular diseases also interact with DMO. Good control of blood pressure reduces the risk of DMO, and cardiovascular disease – particularly ischaemic heart disease or stroke – may be a contra-indication to anti-VEGF injections.^5^ Obstructive sleep apnoea exacerbates retinal damage through intermittent hypoxia, and should be treated if possible. A holistic approach to managing these systemic factors is valuable for effective DMO treatment and prevention.

## Conclusion

DMO is a significant complication of diabetes. It affects vision through breakdown of the blood retinal barrier, leading to retinal thickening and exudates, which affect visual acuity when the fovea is involved. Early detection at primary level is crucial, using the strategies described in this article. Managing DMO requires a multidisciplinary approach involving primary care providers and eye specialists for timely referrals, follow-up care, and tailored treatment plans. Patient education on self-monitoring and lifestyle adjustments is vital to manage risk factors and prevent progression. This collaborative approach is essential for preserving vision and improving patient quality of life.

**From the field: Diabetic macular oedema: a case study from Rwanda**

**Figure:**
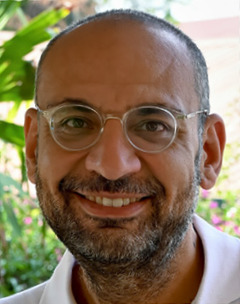

**Michael Mikhail** is an ophthalmologist at Kabgayi Eye Unit in Rwanda.

In 2023, we conducted a review of 140 patients who had received bevacizumab (Avastin) injections for DMO at our clinic. Here is what we found when we analysed their medical records:

- On average, each patient received 3 injections, with some receiving as few as 1 and others as many as 18.
- DMO improved or resolved in 77% of all cases.
- While 55% of patients were treated solely with Avastin injections, others underwent combined treatments. Specifically, 36% received Avastin followed by macular laser therapy, 7% alternated between Avastin and intravitreal triamcinolone acetate (IVTA), and 2% received a combination of Avastin, IVTA, and macular laser.
- After treatment, the average visual acuity improved to 0.4 LogMar (range 0–1.5), compared to the pre-treatment average of 0.68 LogMar (range 0–1.5).
- The central retinal thickness decreased from an average of 408 microns (range 251–800) to 296 microns (range 113–700).
- No serious complications, e.g. retinal detachment or endophthalmitis, were found in this audit.

**Key points to highlight from this audit:**

- The average number of injections per patient was relatively low compared to data from high-resource settings, reflecting some of the barriers discussed in the article on page 12.
- Combining different treatments proved beneficial for some patients with DMO, with the majority receiving conventional macular laser in addition to Avastin. Note that DRCR.net Protocol T demonstrated that despite receiving intensive anti-VEGF therapy during clinical trials, 37–56% of patients with centre-involved diabetic macular oedema still needed macular laser treatment within the first year of treatment.^6^
- While there was improvement in vision, it was modest.
- The multiple withdrawal puncture technique from the same Avastin vial (see page 12) was safe.
